# Clinical Lecture on Diseases of the Pharynx

**Published:** 1906-10-13

**Authors:** Andrew Wylie

**Affiliations:** Assistant Surgeon Central London Throat and Ear Hospital


### Hospital Clinics.

### CLINICAL LECTURE ON DISEASES OF THE PHARYNX.

By Andrew Wylie, M.D.(Glas.), Assistant Surgeon Central London Throat and Ear Hospital.

The following interesting lecture was specially reported for The Hospital, and illustrates the practical character of the teaching given to graduates seeking instruction at this special centre of graduate teaching in London.

The most common diseases the general practitioner has to treat are those belonging to the pharynx. Many troubles and affections, including colds, bronchitis, pneumonia, rheumatism, scarlet-fever, diphtheria, typhoid fever, and syphilis begin with an amount of discomfort to the pharynx

On the other hand, severe maladies like tuberculosis, lupus, syphilis, epithelioma, and the simpler growths—papilloma, fibroma, and polypi—are very common in the pharyngeal region.

Pharyngeal disease is frequently a sequence of occupation, and is found among miners, masons, motorists, vocalists, and public speakers.

### Local Diseases.

*Acute catarrhal pharyngitis* is a very common disease. The symptoms are soreness on swallowing and general *malaise*; the lining membrane of the pharynx, the tonsils, and also the nares, becomes red and inflamed. It manifests itself in various forms. (*a*) Idiopathic, caused by a chill; (*b*) diathetic, when due to gout or rheumatism; (*c*) toxic, from drugs, as mercury and potassium iodide, or infectious fevers like measles and scarlet-fever; (*d*) traumatic, from hot fluids or irritating vapours.

*Acute septic inflammation* of the pharynx, or "hospital sore throat," is another manifestation of local mischief. The symptoms are general *malaise*, rigor, rise of temperature, pain and difficulty in swallowing. The mucous membrane becomes hyperæmic, swollen, and œdematous, and these conditions, extending to the submucous tissues, give rise to suppuration. Some cases may spread rapidly until suppurative cellulitis ensues, extending to the larynx and trachea and ending fatally.

In order to treat this condition effectively the patients must be fed as well as possible, iron and strychnine administered internally, and local antiseptic soothing solutions employed as a mouth-wash.

A third local disease is *chronic pharyngitis*, which may be the result of (*a*) simple catarrh; (*b*) nasal obstruction; (*c*) fevers; (*d*) gouty diathesis; (*e*) exposure to dust or irritating fumes; (*f*) excessive use of tobacco or alcohol; (*g*) dyspepsia, constipation, portal congestion; (*h*) anæmia; (*i*) neurotic temperament in females.

In this condition the patient complains of weakness and discomfort and a dragging sensation in the fauces, with the continual "hawking" up of mucus. The voice gets tired and an irritable cough ensues. The mucous membrane is in some cases congested and the lymph follicles hypertrophied; in other cases it is dry and pale-looking and there is a diminution of lymph follicles. This latter condition is called *pharyngitis sicca*, and is usually associated with nasal disease.

In treating this condition, if gout, anæmia, dyspepsia, hepatic disease are present, these should be attended to. A course of saline water is usually recommended at some natural spring. In this country there are many good mineral springs, as at Buxton, Bath, and Strathpeffer. The wonderful success of the fashionable continental springs is not due to any great virtue in the waters, but to the thorough way in which the treatment is carried out by patients and doctors at these places. Besides the mineral springs at Aix-les-Bains or Homburg, a thorough system of local treatment is recommended. Mild astringents like potassium chlorate or sodium bicarbonate, or, in more severe or granular cases, painting the pharynx with sulphate of zinc (20 grains to the ounce), perchloride of iron, or Mandl's solution (iodine 10 grains to glycerine 1 oz.) must be resorted to. If large granular lymph follicles are present they can be treated quickly by the galvano-cautery. Vocalists, clergymen, and other public speakers should be taught how to rest the voice and use it properly.

A fourth condition is *acute tonsillitis*, usually caused by changeable weather. It appears most commonly in the hot season of the year, when there is a sudden change of temperature. In many cases of this disease the Klebs-Loeffler bacillus is present, and such cases at first should be treated as diphtheria until the contrary diagnosis is proved. In lacunar tonsillitis the mucous membrane of the tonsil becomes inflamed, with patches of yellowish exudation from the crypts. Soon the deeper tissues are involved and the swelling of the tonsil becomes very severe. This condition is termed *parenchymatous tonsillitis*. When the connective tissue in front and above the tonsil becomes inflamed the term *peritonsillitis* is applied, and when suppuration takes place a *peritonsillar abscess* is the result.

The symptoms depend on the degree of inflammation. It commences with pain in the throat, aching of the limbs, and a feeling of *malaise*, a slight rigor, rise of temperature, constipation, and a coated tongue. Often a slight erythematous rash appears on the surface of the body, resembling the scarlet-fever rash. This is fairly common a few days after the operation of tonsillotomy, and if the surgeon is not familiar with such a condition considerable anxiety often ensues. I have known several cases of children with a slight rash after the removal of tonsils being diagnosed as having contracted scarlet-fever, and I remember one such case which was removed to an isolation hospital. When the inflammation of the tonsil extends there is considerable pain in the ears and great difficulty in swallowing. The sub-maxillary glands become infected, and the saliva is profusely secreted and allowed to flow from the mouth. This inflammation, extending to the peritonsillar tissue, forms a large smooth swelling in front of the tonsil. If fluctuation is elicited, pus is present, and should be removed at once. The usual way is to anæsthetise the swelling with a 10-per-cent. solution of cocaine. Use a guarded knife—that is, a knife with no long blade which might injure the tongue or lips if the patient moved, but one with a point only. The point of incision is well above the tonsil, and should be made in the lateral direction, nearly at the level of the base of the uvula. One free incision should be made, and the wound subsequently opened up with a pair of sinus forceps, this ensuring free exit of the pus and subsequent drainage. Various knives and instruments may be employed for this operation; a very ingenious appliance is composed of a spring which, when pressed, reveals a concealed knife for the incision and dilatation of the wound. The drawback is it cannot readily be taken to pieces for sterilising purposes.

The treatment of acute tonsillitis is not very complicated. Sodium salicylate is given in fairly large doses every three hours, with gr. ij. to gr. iv.

The fauces should be painted with guaiacum and gargled with potassium chlorate or with a carbolic acid solution. Small pieces of ice are given to suck, and the strength maintained by beef-tea, milk, and brandy. As soon as the acute symptoms begin to disappear perchloride of iron in large doses is recommended.

I do not advise removal of inflamed tonsils, for the general health being lowered and the tonsil probably suppurating, it is inadvisable to form any extensive raw surface.

*Chronic enlargement of the tonsil* is often the result of repeated acute attacks, occurring in strumous children or amongst people inhabiting damp houses or moist districts.

The patient in the trouble is subject to acute or sub-acute attacks of tonsillitis. Enlargement of the tonsils causes difficulty in breathing. Adenoid growths are usually present in the naso-pharynx. The lingual tonsil may be enlarged, and the patient generally breathes through the mouth and snores at night. The hearing becomes affected, the voice becomes altered, and the difficulty in breathing interferes—especially in children—with the development of the chest, and what is called "pigeon breast" is a common result. The patient grows up weak mentally as well as bodily. The tonsil may atrophy, but before that time comes, as I have explained, the child's health has become impaired. The simple operation of removing the tonsils obviates all these consequences.

The treatment indicates the removal of the tonsils and adenoid growths whenever they are present. The plan adopted at the Central London Hospital is admirable. The anæsthetic generally administered is chloride of ethyl, which gives the surgeon more time than nitrous oxide gas: it gives a thorough anæsthesia for a few minutes; but ether or nitrous oxide and ether are excellent in this position, and on several occasions I have had chloroform given with no bad effect. Different surgeons prefer different tonsillotomes. I prefer Heath's, which is easily taken to pieces to be cleaned. In some cases, where the tonsil is very large, or in an adult, where the hæmorrhage is often troublesome after using a tonsillotome, I use a wire écraseur or take the tonsil out piecemeal by means of punch-forceps. Severe hæmorrhage after tonsillotomy is rare, but slight hæmorrhage is common. It usually comes on several hours after the operation, and is due to: (*a*) An abnormality of the blood-vessels, (*b*) hæmophilia, (*c*) a small piece half cut through and left, (*d*) excessive retching and vomiting. If severe, the bleeding point must be caught by artery-forceps or touched with the galvano-cautery; but as a general rule ice to suck and complete rest curtails it.

*Hæmorrhages* occur from the pharynx from several causes, upon which the treatment depends. The causes are (*a*) general debility from albuminuria, diabetes, and fevers; (*b*) acute inflammations; (*c*) ulcerations, as tubercular, malignant, and specific; (*d*) after excessive mercurial treatment; (*e*) enlarged varicose veins or hepatic disease; (*f*) traumatic, from a needle, pin, gun-shot wound, or, as in a case I saw lately, where a man fell on the point of his umbrella and it entered his pharynx.

*Retro-pharyngeal abscess* is another local disease of the pharynx, although it is usually associated with a general strumous condition, chiefly seen in young children. It may simply be a swelling and suppuration of the cellular tissue on the posterior pharyngeal wall, or it may be due to caries, generally tubercular, of the upper cervical vertebræ. The symptoms are difficulty in respiration, causing a croupy sound and slight dyspnœa. The head becomes fixed, there is swelling of the glands at the angle of the jaw, and pain when pressing on the head. On examination a bulging is seen on the posterior surface, and often fluctuation will be felt with the finger. The treatment is to evacuate the pus. An incision is made into the most prominent part with a guarded knife. Immediately the knife is withdrawn the child should be turned "upside down," with the head low, to keep the pus from running into the larynx or œsophagus. I had a case lately, in a young girl aged twenty-one, which I opened from the posterior border of the sterno-mastoid muscle, and with forceps made an opening into the posterior wall of the pharynx. (*Lancet*, April 14, 1906.)

Simple and malignant growths are common in the pharynx and on the tonsils. The usual ones are papilloma, adenoma, fibroma, and polypi hanging from the naso-pharynx.

### General Diseases.

General diseases of the pharynx include diphtheria, syphilis, tuberculosis, malignant disease, mycosis, lupus, sensory and motor neuroses.

### Diphtheria.

Diphtheria as a disease was described by physicians in the second century, but it was not until 1821 that any definite written statement concerning it was found. The disease prevails in every country and town, but more so in country districts and in dry weather. It attacks children, chiefly from one to five years old, but may affect any age. Diphtheria is fortunately rare at a throat hospital clinic. General practitioners encounter most cases of diphtheria. It is a general disease with distinct local manifestations in any mucous membrane, but usually manifest in the fauces, sometimes in the nose or larynx. Diphtheria is due to a distinct bacillus called the Klebs-Lœffler. An inflammation of the throat where the bacillus is found is diphtheria, but, on the other hand, the bacillus may be found in many throats where there are no signs of inflammation or membrane, and these are not described as diphtheria.

### Symptoms.

The disease often resembles acute lacunar tonsillitis, and, if the membrane is in patches, it resembles acute follicular tonsillitis. The diagnosis depends on the bacteriological examination and several prominent symptoms of diphtheria, say prostration and drowsiness—a symptom which is not often referred to in text-books.

The disease is ushered in by a slight chill and sore throat; the temperature is never very high, and may even be normal; the pulse weak, and the ensuing prostration severe. These general symptoms advance quickly, and the prognosis depends greatly on the degree of prostration. The local symptoms are, first, a soreness in swallowing and symptoms of acute pharyngitis; the glands at the angle of the lower jaw swell, and in a few days patches appear on the tonsils and uvula. These are thin and translucent at first, but become united to form the characteristic tough, greyish-white false membrane covering the uvula, soft palate, tonsils, and pharyngeal walls. This is a true diphtheritic membrane, firmly fixed to the fauces. If it is scraped off, a bleeding surface results.

### Diagnosis.

Diphtheritic throats may be confused with (1) follicular tonsillitis; (2) scarlet fever before the rash appears; (3) erysipelas of the fauces; (4) enteric fever, which is often ushered in by tonsillitis; (5) mumps; (6) septic sore throats. In any doubt the treatment remains identical in every case.

Complications and sequelæ are the chief things to fight against. These are briefly stated as follows: (1) Prostration, both during the disease and afterwards, due to absorption of septic poison. (2) Cardiac failure is one of the most fatal complications. (3) Paralysis may occur early or later, and is a common result. The muscles of the soft palate are most commonly affected; it loses its power of sensation and movement, and liquids regurgitate through the nose. The paralysis often affects the legs, the arms, the ocular muscles, the abductor laryngeal muscles, and even the diaphragm. (4) Inflammation of the middle ear (otitis media) is a common occurrence, and (5) Albuminuria and pneumonia are well-known complications.

### The Treatment.

The treatment is definite on the first real suspicion of diphtheria. The suspicion is increased by the occurrence of sore throat in places where diphtheria has been known to exist. Antitoxin serum must at once be injected. There is no excuse for delay; 2,000 units should be injected at once. If the case turns out not to be true diphtheria, this injection will have done no harm. On the other hand, the injection has, in many cases, done good even in a simple follicular tonsillitis, but especially in the sore throat of scarlet fever. Within the first twenty-four hours of the disease 2,000 to 3,000 units repeated may be enough, but if the disease is not diagnosed at once the antitoxin treatment must be pushed—8,000 to 10,000 units repeated. It is better to give an overdose than too small a dose. The syringe should be made so that all parts can be sterilised. There should be no tubing between the syringe and the needle; the latter is better fixed direct to the syringe, which should hold 8,000 units at least. The abdominal subcutaneous tissue is the most convenient place for injection; the skin over the point to be punctured should be well washed and rendered aseptic. The injection causes practically no pain, and there should be no abscess or irritation afterwards if aseptic details are attended to. Vernot and Behring state that the sooner the antitoxin is administered the less paralysis supervenes, though other writers state that serum treatment has increased the amount of post-diphtheritic paralysis.

The explanation really is that those cases which live to develop the nerve lesions after being cured by the antitoxin would probably have died and been lost to sight or statistics if antitoxin had not been administered.

The point to be emphasised is the early administration of antitoxin. Do not wait for "swabs" to be taken, sent to a pathologist and reported on. Inject 3,000 units of antitoxin in every suspected case. If it is only a tonsillitis or scarlet-fever sore throat it will do no harm; in fact, several clinical observers have declared it has done good, and I know of several medical men who inject a small dose into every scarlet fever case.

The early injection prevents the diphtheritic toxin from combining with the body cells. When once combination with the tissues has occurred, large doses of antitoxin do not have the same power. The dose of antitoxin will keep more of the poison from being absorbed, but the amount of poison already absorbed must run its course and do its amount of mischief. Early administration, therefore, will act as an antidote; later administration only as a remedy.

In nasal and laryngeal diphtheria the injection should be administered even earlier.

Diphtheria, therefore, if suspected, should have 2,000 units injected at once; next day, if still suspicious, 4,000 units; the third day 6,000 units, and even a second dose; on the fourth day, 8,000 to 10,000 units, and repeated in six hours. If there is no result after this then the diphtheritic poison has got well absorbed; and in that case I would advise intravenous injection of antitoxin.

As regards local treatment, douching the nose and throat with a solution of boracic acid or very weak carbolic lotion is most soothing to the patient. A little powdered orthoform and resorcin blown on to the pharynx alleviates the acute symptoms. The general health must be attended to. Wine, brandy, beef-tea, milk, and stimulating foods must be literally poured into the patient to overcome the acute prostration and to ward off paralysis.

### Syphilis.

*Inherited Syphilis* in the pharynx appears as secondary and tertiary lesions. Usually Hutchinson's teeth and keratitis are present, singly or together. A primary sore is rare in the pharynx, but has been found on the tonsil. As a rule it is not diagnosed as syphilis until the secondaries appear. A case came to this hospital a few years ago with secondary syphilis due to infected instruments (*Lancet*, 1902). Secondary syphilis of the pharynx is as a rule easily diagnosed. There is a bright reddish symmetrical hyperæmia, well confined to the soft palate. Bluish-white patches appear on the tonsils, faucial pillars, pharynx, gums, and lips. These patches are nearly always bilateral and symmetrical, called by Hutchinson "The Dutch garden symmetry." Other symptoms of syphilis are present, such as the rose-coloured spots on the chest and abdomen. It is differentiated from diphtheria and septic sore throat by the pain being absent or else very slight.

In treating it, push mercury until salivation threatens, then diminish it or stop it for a few days every fortnight, and continue it again. The best form is a pill made of hydrarg. cum creta gr. ij. three times a day, and to obviate any intestinal trouble a small quantity of Dover's powder should be added. If the case has not been under treatment for some time it is often best to give the liquor hydrargyri perchloridi in one-drachm doses, combined with potassium iodide gr. x. The throat should be gargled with borax and potass. chlorate, the teeth and gums carefully washed every few hours, and the general health attended to. Tertiary lesions are often found in the pharynx, especially in the soft palate. They come on without pain or discomfort, and continue until deep ulceration and loss of tissue result. Tertiary lesions are very rapid, and extensive destruction of the soft structures and necrosis of the bone may occur without the patient's knowledge. It is, therefore, highly important to diagnose a case of gumma of the pharynx early. The soft palate is usually the part affected, and the first sign may be merely a want of movement. Careful examination with the post-pharyngeal mirror shows an injected boggy mass, which, if undetected, would soon break down and lead to a perforation difficult to heal and causing great discomfort on speaking.

In tertiary syphilis the treatment is potassium iodide, beginning with small doses of gr. 10 and increasing the dose to gr. 20, 30, 40, and even 50., unless great depression and prostration result. Many patients can take large doses of potassium iodide without any bad results when small doses would cause severe coryza, eruptions, and depression. It is a good plan to add liquor hydrargyri perchloridi in one-drachm doses to the potassium iodide and aromatic spirits of ammonia. A good prescription is: —

| | |
|---|---|
| Potass. iodid. ... ... ... ... | gr. xv. |
| Liq. hydrarg. perchlor ... ... ... | dr. i. |
| Spir. amm. arom. ... ... ... | dr. i. |
| Infus. quass. ... ... ... ... | dr. $\frac{1}{2}$ |
| Aqua ad ... ... ... ... ... | ℥j. |

In using potassium iodide or mercury it is most important to wash the mouth with potassium chlorate and thoroughly clean the teeth.

Other forms of treatment are: (*a*) *The Inunction of unguent. hydrarg.*, about $\frac{1}{2}$ to 1 drachm, rubbed into the axilla or groin every night. (*b*) *Hypodermic injections of mercury*. I have found the hydrarge benzol most efficacious, no abscess forming at the seat of injection. The treatment of syphilis must be continued long after the patient is apparently well, or else sooner or later other manifestations of the disease will appear, not perhaps in the pharynx, but in other organs of the body.

A rule is set down in text-books that after two years' treatment a patient is cured, may marry, and have healthy children. A discussion is going on at present proving, I think correctly, that syphilis is transmitted to the offspring even after two years' treatment.

